# The American Society of Dental Surgeons, and the Dental Register of the West

**Published:** 1848-07

**Authors:** E. Taylor


From the New York Dental Recorder.

### THE AMERICAN SOCIETY OF DENTAL SURGEONS,

### AND THE DENTAL REGISTER OF THE WEST.

We have already alluded to our spirited cotemporary, the Dental Register of the West. Since the publication of our last, the April No. has come to hand, and among several excellent articles, we noticed particularly the "COMMENTS *on the actions of the American Society of Dental Surgeons, by* B. B. BROWN, M. D., D. S., *St. Louis Editor.*" The previous number of the Register also contained an article reviewing the proceedings of the American Society for 1847, written by its Cincinnati editor, Dr. James Taylor. Both of these gentlemen were members of the American Society, and both refused to sign the odious protest against Amalgam which the Society ordered in 1845. The St. Louis editor, for this cause, was at the last meeting of the Society, formally expelled; and the case of the latter, with several others, was laid over for another year.

Neither of these gentlemen, although now very indignant at the action of the Society, cared enough about the subject last summer or the summer before to attend the annual meetings—had they been present, they might have had an opportunity of "vindicating themselves in person," while their votes would have "preserved the Society from the worse than Vandalism which characterized its proceedings." Had they been present, they would have known better than to accuse any of those members, who have from the commencement opposed this bigoted and contemptible crusade against the freedom of opinion and of action guaranteed to the members of the American Society by its own constitution, of "halting and wincing," or of "sacrificing their principles as previously expressed by themselves," or "their self-respect by succumbing to the imperious action of that small and factious majority then present."

We do not accuse either of the editors of the Dental Register, of intentionally misrepresenting the course of any members. As they were not present, their only means of knowledge upon the subject must have been derived from hearsay, by no means the best authority; or from the imperfect, and in some respects errone-

3

ous report of the secretary published in the Journal of the Society.

The only members of the Society whom the Dental Register can refer to, as compromising their principles to retain their membership, were Dr. B. Lord, of this city, and the editor of the Dental Recorder, and if the Secretary of the Society had seen fit to publish the "pledges" of these gentlemen, as he surely should have done, it would have been known that they are still as strongly opposed to the protest of the Society as they ever were, or as the St. Louis editor himself now is—in fact that they have never signed that protest, and never intend to, any more than Dr. B. B. Brown, or Dr. J. Taylor.

We are unwilling to occupy the pages of the Recorder with personal matters of our own, but, as in this case another stands accused with us of "sacrificing his principles," and as it is an act of justice which we would not refuse to him, we are disposed to lay our own case before the Profession that all may judge of the "halting and wincing" for themselves.

In 1845, we were reported by the inquisitorial committee, appointed to inquire into the practice of members, as being among the amalgam dentists. We opposed the action of the society upon this subject, at that meeting, as a violation of professional privilege, unjustifiable and undignified on the part of the society; and it was not until we were persuaded by several of our professional friends, to compromise the matter by relinquishing the use of amalgam, that the action of the society to prevent its great abuse throughout the country, might be unanimous, that we consented, on the eve of the last day of the session, to do so. As we were prevented by professional engagements from attending the meeting on the last day the following letter was sent to the President and read before the Society.

*Mr. President and Members of the American Society of Dental Surgeons.*

When I first learned that this Society had decided that, for the purpose of suppressing malpractice in dentistry, each and every one of its members should pledge himself by his signature not to use the silver amalgam in any case whatever. I did not see how I could conscientiously comply with its requisitions, for the following reasons:

1st. I considered it an unwise and arbitrary measure for this society to first pass a resolution that a certain course of practice which many of its members were pursuing, was malpractice, and then to discipline those members without proving that injury had been done to their patients by such practice.

2d. From circumstances which had come to my knowledge, it appeared to me like espousing the quarrels of some members of this society, who had to contend with rivals who were in the practise of using that proscribed article which, if true, I considered incompatible with the high and dignified stand that I wished this society to take.

3d. I could not but consider it as an attack upon the practice of many members of this society, who were honestly using the amalgam for the benefit of their patients.

4th. I doubted whether it would produce the desired effect—that of suppressing quackery and imposture; and feared that the many educated and honest dentists, not members of this society, who are daily using the mercury and silver would plead to their patients, and at the bar of public opinion, which I have always thought was in favor of the amalgam, that the society of dentists was proscribing and persecuting them because they would not become members and submit to be dictated to by the society.

5th. I had myself used the article (Amalgam) occasionally, for more than eight years, never from choice, but only when I could not use either gold or tin, without great danger of splitting the tooth, and in all of these cases I had used it experimentally, watching the effects of the operation with great care, and I can truly say that I have never seen any bad effects resulting from its use, which I have not seen from the use of both gold and tin; but, that, in many cases, it has preserved the teeth, strengthening and making them useful organs of mastication for many years.

Entertaining these views I felt exceedingly unwilling to abandon the use of the amalgam, but as many of the members of this society who have used the article more than I have, and for whose opinion I have the highest regard, have decided to use it no more, and as I do not wish to impose the slightest obstacle to the success, union and harmony of the society, I cheerfully consent to do the same, hoping that all the members will see the importance of making mutual concession if they would be a happy, useful and prosperous society. [SIGNED.] C. C. ALLEN.

After this letter was received by the society, the resolutions ordering the protest were passed, and the first intimation which we had of these proceedings was the receipt of the blank protest, sent by the Secretary for our signature, from which we instinctively revolted. Soon after this we gave our views in opposition to the whole proceeding in the American Journal. Through the session of the society held in 1846, we continued with the minority against the arbitrary mandate of the society, but neither of the editors of the Dental Register were there to assist us. It was at this meeting that the Vandals overrun and trampled down the 6th article of the constitution, the injustice and folly of which has been exposed by the Cincinnati editor.

Here then the matter rested until the meeting of the society in 1847, when owing to the absence of the lukewarm, and the presence of a few dough faces, the Vandals again had it all their own way, by a majority of one. After resolving that they would not expel any member who was not in the practice of *using* or *recommending* the use of amalgam for filling teeth, those present who had not signed the protest were called upon to state whether they used the article or not. As Dr. Lord and myself had abandoned the use of amalgam in our practice two years before, there seemed no good reason why we should not now say so to the society. To have refused would have looked like a foolish desire for martyrdom on our part. We therefore offered to furnish the society with a copy of the "pledge," as the secretary calls it, which we had given it two years before, and which the secretary informed us he had not then in his possession. The following was therefore handed in without the words printed in italics.

*Saratoga Springs, August 5th,* 1847.

Two years since I felt exceedingly unwilling to abandon the use of amalgam, but as many of the members of this society who have used this article more than I have, and for whose opinion I had the highest regard, then decided to use it no more, *nor to encourage its use,* and as I did not wish to impose the slightest obstacle to the success, union and harmony of this society, I cheerfully consented to do the same, hoping that all the members would feel the importance of making mutual concession, if they would be a happy, useful and prosperous society. [SIGNED] CHAS. C. ALLEN.

This the secretary says was not accepted, but "Dr. Allen was requested to draft one in accordance with the said resolution."— We accordingly added the following, "That concession I consider binding upon me so long as I remain a member," and handed it in as our ultimatum. The pledge of Dr. Lord was similar in phraseology to our own. We had previously intimated to Dr. Keep, that if he tendered his resignation, we should do the same; but as we had enlisted for the war and wished to fight it through, and assist in decently burying the dead, we did not tender our resignation until some time after the termination of the session. It is now in the hands of the secretary, and here ends our connection with the American Society of Dental Surgeons.

---

MESSRS. EDITORS:

In the June No. of the New York Dental Recorder, I see the Editor evinces a commendable sensitiveness of the passing notice I gave him in the brief review I made of the proceedings of the American Society of Dental Surgeons on the Amalgam controversy. I should be very far from designedly misrepresenting the course of any one, but my *only* means of knowledge was through the report of the organ of that society, which I had not seen represented as incorrect. That No. of the Journal is not now at hand, but I think if the Dr. should refer to it, that he will regard that report as sustaining my remark.

I am sorry to see the Dr. placed in a false position, as I had been led to form a high opinion of his character, and have *since* learned that by his "coolness and intrepidity" he did nobly battle to the last, and refused to succumb, and am glad if my remark has been the occasion of his placing himself in his right position.

The Dr. is in error in regarding me, the Cincinnati Editor of the Register, as being before the Society. I had several years since withdrawn from active membership in that Society, regarding my location as probably precluding my ever again participating in the meetings, and wishing to devote my influence to the advance of the interests of the profession in the west. I, however, continued to feel a deep interest in the action of our eastern brethren, and continued to take the Journal until three of the last five No.'s due, failed to reach me, and having so much trouble to obtain the No.'s that had failed to reach me, one of which, indeed, I have never been able to obtain, that I thought it best to discontinue it. My present views are against the use of the Amalgum, as they ever have been. My opinions were formed from the report of others; and as I regarded, the deductions of science. I have used it experimentally in a few cases which I have watched with some interest, and although several years have passed, nothing alarming has occurred. My own view of the case is, that every one should fully and intelligently make up his mind as to the practicability of its use, and do whatever he conscientiously believes the interest of his patient demands. If a connection with any society prevents him from doing the most ample justice to his patient, that connection should be renounced at once.

E. TAYLOR.

